# C-Reactive Protein as a Predictor of Severe Respiratory Complications in Measles

**DOI:** 10.3390/medicina60071031

**Published:** 2024-06-24

**Authors:** Lidija Popović Dragonjić, Aleksandar Ranković, Milica Ćosić Petković, Maja Cvetanović, Jelena Miladinović, Andrija Jović, Jovana Tomić, Nikola M. Stojanović

**Affiliations:** 1 Clinic for Infectology, University Clinical Center Niš, 18000 Niš, Serbia; drarankovic@gmail.com (A.R.); cosic.milica93@gmail.com (M.Ć.P.); maja.majcan@hotmail.com (M.C.); miladinovic.jelena.ni@gmail.com (J.M.); dr.risticjovana991@gmail.com (J.T.); 2 Department of Infectious Diseases and Epidemiology, Faculty of Medicine, University of Niš, 18000 Niš, Serbia; 3 Clinic for Dermatology, University Clinical Center Niš, 18000 Niš, Serbia; dr.andrijajovic91@gmail.com; 4 Department of Physiology, Faculty of Medicine, University of Niš, 18000 Niš, Serbia; nikola.st90@yahoo.com

**Keywords:** measles, C-reactive protein, pneumonia, complications, chest X-ray

## Abstract

*Background and Objectives*: Even though measles is easily prevented by vaccination, infection outbreaks are not rare. Infection carries a great risk for pulmonary complications, which are sometimes hard to predict, especially in a group of outpatients. This study aims to evaluate the association between serum CRP changes and the severity of respiratory complications in the group of inpatients treated for measles. *Materials and Methods*: A total of 207 patients admitted and treated at the Clinic for Infectious Diseases, University Clinical Center, Nis, for measles infection were included in the analysis. The data collected from the patients’ medical records included demographic characteristics, disease duration, blood and serum biochemical analysis, general measles-associated symptoms, and disease outcome. *Results*: Results of the study revealed that there are almost no differences in the clinical presentation of patients with measles and those complicated with pneumonia. The examined CRP changes are found to correlate with the observable degree of pneumonia; however, they do not correspond to the changes visible in chest X-rays. *Conclusions*: CRP changes in the serum of patients with measles with mild clinical pictures could be a potential predictor for the development of some pulmonary complications.

## 1. Introduction

Measles is a highly contagious viral and vaccine-preventable disease. More than 100,000 patients die annually from measles. The measles virus is transmitted via airborne respiratory droplets. It is important to note that the measles infection can vary from region to region and over time, and public health organizations, such as the World Health Organization (WHO), continuously monitor measles cases and work towards maintaining high vaccination rates to prevent its resurgence [1]. Measles infection goes through a prodromal phase (elevated temperature, cough, coryza, conjunctivitis) and a rash phase, occurring a few days after the appearance of increased temperature, in the form of a maculopapular rash. Koplik’s spots, small white papules, appear on the buccal mucosa during the prodromal phase and often disappear before the rash appears. In people who have not developed measles complications, recovery begins within a week of the appearance of the rash [2]. Measles can affect many organ systems in the human body, and pneumonia is one of the complications associated with the highest mortality rate [3]. Adult age bears an increased risk for severe respiratory consequences, and one of them includes respiratory failure [4]. Some other risks might include a compromised immune system, malnutrition and vitamin D deficiency [4].

After the initial (prodromal) phase, patients with measles develop respiratory symptoms and pneumonia, have difficulty breathing, rapid breathing, and hypoxia [3]. Further progression to respiratory failure includes severe dyspnea, cyanosis (bluish discoloration of the skin due to lack of oxygen) and marked decreased oxygen saturation levels. The intensity and fast progression of symptoms might need to be taken care of in the intensive care units where mechanical respiratory support is applied [3]. Since this progression to pneumonia and in the severity of the disease is rapid, it would be good if there were some clinical parameters that could be used for monitoring.

C-reactive protein (CRP) levels in the serum of patients with measles increase concurrently with the onset of the rash. It has been noted that patients with measles complicated by pneumonia have prolonged elevated serum CRP values and that a second peak in CRP elevation can occur in patients with measles encephalitis [5]. The results of a study that analyzed 72 children with measles showed that mean CRP values were significantly lower in measles without complications than in severe forms of measles with complications, from which the conclusion was drawn that CRP values can indicate the course and possible development of measles complications [6].

Since CRP is a non-specific marker of inflammation, elevated values of this parameter do not have to point to bacterial infection exclusively. In fact, moderately elevated CRP values can also be found in severe or untreated viral infections, such as viral infections of the upper respiratory tract or lower respiratory tract [7,8]. Values of CRP can also increase significantly in viral pneumonia; thus, without additional analyses that would confirm the bacterial etiology of the disease, prescribing antibiotics can be of no use [9]. It has also been proven that a rapid and substantial increase in CRP predicts the development of acute respiratory failure in COVID-19 patients [10].

The present study aimed to evaluate the levels of CRP in severe measles complicated by primary morbillous pneumonia with various degrees of respiratory insufficiency and to determine its potential utility as a predictor of the severity of morbillous infection.

## 2. Materials and Methods

### 2.1. Study Design and Data Collection

The measles outbreak took place in Nišava and Toplica districts in south-eastern Serbia for the period from 20 November 2017 until 8 May 2018. There were 1473 outpatients examined for measles by a specialist at the Clinic for Infectious Diseases, Niš, Serbia, out of which measles were confirmed in 1240 patients. The study protocol was presented and approved by the Medical Ethical Committees of the University Clinical Center Niš in Serbia (decision number: 28355/13) and the Faculty of Medicine University of Niš (Niš, Serbia). Informed consent was obtained from all patients before the commencement of the study.

The diagnosis of measles was established based on three criteria:

(i) clinical manifestations (cough, conjunctivitis, coryza, characteristic Koplik spots, appearance of a maculopapular rash);
(ii) epidemiological questioner;
(iii) serological analysis (positive measles-specific immunoglobulin M (IgM)).

Out of a total of 224 hospitalized adult patients, 207 adult patients were further analyzed and processed, 130 females and 77 males, since these patients’ complete data from their medical histories were available. The data collected from the patients’ medical records were as follows: (i) demographic characteristics (age and gender); (ii) disease duration; (iii) whole-blood and serum analysis; (iv) general measles-associated symptoms; and (v) disease outcome.

In this study, patients with chest X-rays suggesting viral pneumonia (bilateral perihilar peribronchial thickening and interstitial infiltrates) and with signs of adult acute respiratory distress syndrome (ARDS) (asymmetrical pulmonary ground-glass opacification on computed tomography) were included. Respiratory insufficiency was monitored through the Hb saturation levels (SpO_2_) measured initially via a pulse oximeter, and afterwards with blood gas analyses. The study group of patients with pneumonia was initially divided into two groups based on respiratory insufficiency. Furthermore, based on the extent of hypoxemia, the group of patients with respiratory insufficiency was divided into 4 subgroups according to previously given criteria [11], specifically into those with low-to-normal hypoxemia (SaO_2_ = 95–100%, PaO_2_ approx. 90 mmHg), mild hypoxemia (SaO_2_ = 90–94%, PaO_2_ approx. 80 mmHg), moderate hypoxemia (SaO_2_ = 85–89%, PaO_2_ approx. 60 mmHg), or severe hypoxemia (SaO_2_ less than 85%, PaO_2_ less than 50 mmHg).

A total of 207 patients were analyzed in detail and, based on the results of their laboratory parameters, general clinical presentation and additional diagnostic methods, conclusions were drawn. The sample size can be considered adequate, since 1/6 of the total morbilli positive patients, and around 92% of all hospitalized patients, were included in the study.

### 2.2. Serum CRP Analysis and Monitoring

Serum CRP values were analyzed in patients without bacterial infection, confirmed either through the absence of any clinical manifestations and/or the negative cultivation of nasal or throat swabs, sputum, urine samples, or fecal samples. Values of CRP were measured using an automated biochemical analyzer (Olympus AU680, Beckman Coulter, Los Angeles, CA, USA), which was also used for the determination of other standard serum biochemical parameters such as lactate dehydrogenase levels (LDH).

The connection between serum CRP values was performed on a stratified sample of subjects with pneumonia, where the CRP serum values were increased less than 2-fold (category 1) between 2 and 3 folds (category 2), between 3 and 4 folds (category 3), and equal or larger than 4 folds (category 4). Since the association between CRP values and AMP in patients with measles was examined in this study, at the commencement of this study, we included only the patients without bacterial co-infection.

### 2.3. Statistical Analysis

Data are presented as frequency distributions expressed as percentages or median/mean values. The normality distribution of continuous variables was confirmed by the Kolmogorov–Smirnov test. The comparison of mean values was done by either Student’s *t*-test or the Mann–Whitney U test, depending on the variables’ distribution. Categorical variables were compared using a Chi-squared or Fischer’s exact test, depending on group size. The statistical package SPSS (version 21.0, IBM Corp, 2012; Armonk, NY, USA) was used for all statistical data processing [12]. A two-sided *p* < 0.05 was considered to be statistically significant.

## 3. Results

Out of the total number of patients (*n* = 207), 77 of them were males, while 130 were females, with an average age of around 36 years (Table 1). Patients developed symptoms of infection roughly 5 days prior to hospitalization and spent around 7 days in hospital (Table 1). As many as 98 patients had viral morbillous pneumonia (47.3% of all statistically analyzed and hospitalized patients), and 33 were men, while 109 did not develop pneumonia (Table 1). Slightly longer hospital duration was in patients with morbillous pneumonia, and the disease duration and prehospital symptom duration was almost identical to the total population and those without pneumonia (Table 1). Two patients, both female, progressed from viral pneumonia to ARDS, and one case of ARDS had a fatal outcome.

Blood parameters where maximal WBC and neutrophile abundance were measured were found to be slightly altered compared to the reference values in the total sample and in the group of patients that did not develop pneumonia (Table 2). At the same time, LDH activity was high above the uppercut value, as were the CRP values prior to and during hospitalization (Table 2). In the group of patients with pneumonia, the same increased values were more pronounced than in the total sample (Table 2).

Observed clinical parameters in patients with morbilli infection are given in Table 3. The average fever duration was 7.5 days, and temperature varied between 37.2 to 41 °C. Radiographic changes in the lungs were noted in almost 50% of patients, and phenomena observed in a small number of patients included mucosal changes such as Koplik spots and enanthem (Table 3). A large number of patients experienced dry cough (82.6%), followed by the feeling of asphyxiation and sore throat, 45.9% and 42%, respectively. In the group of patients without pneumonia, a very small number of subjects experienced asphyxiation, but dry cough and sore throat were present (Table 3). The total clinical sample comprising 207 patients had around 16% of patients with other somatic comorbidities, among which hypertension was the dominant one (Table 3). Similar results were obtained in the group of patients with pneumonia (Table 3).

In a certain number of patients (*n* = 77), CRP values were closely monitored during hospitalization, and the dynamic of change (as described in the Materials and Methods section) was calculated. According to an increase in CRP, only two patients were categorized in category No 1, 44 were categorized in category No 2, 14 of them were in category No 3, and 16 of them were in category No 4. 

Chi-square test results revealed a significant association between the changes in CRP and the intensity of pneumonia (Table 4), indicating that, with the intensification of pneumonia, the serum CRP increases as well. The association (Spearman’s correlation coefficient) between the four mentioned categories of CRP changes and pneumonia with different extents of hypoxia. In a total sample, a statistically significant correlation was detected between changes in CRP levels and hypoxia extent (R = 0.543; *p* = 0.0001), suggesting that, with the occurrence of hypoxia or worsening of the state, the CRP levels increase several folds.

Using the Chi-square test, the association between changes in CRP and chest X-ray changes was analyzed. The obtained results showed no significant difference between the two studied parameters (Table 5).

## 4. Discussion

CRP is an acute phase reactant that is released in the process of inflammation during infection with various infectious agents, including infection with various viruses. If serum CRP levels increase in the context of viral lower airway infection, the primary reason for this elevation is often the emergence of bacterial co-infection. Occasionally, the rise in CRP can be attributed to the exacerbation of virus-induced tissue damage. In rare cases, other inflammatory complications may also contribute to the elevation of CRP levels [9]. The results of a meta-analysis conducted by Canadian researchers showed that CRP values in 230 patients who developed complications were statistically significant between the groups of patients with pneumonia not requiring hospitalization and the groups of patients with pneumonia requiring hospitalization and intensive care, as well as those who had a fatal outcome [13]. Also, the increased value of CRP in itself does not indicate the existence of infection with the flu virus, nor does the degree of increase in CRP, which might indicate inflammation and clinical presentation intensity, correlating with influenza A infection. However, signs of a more serious clinical picture in H1N1 influenza are associated with elevated CRP levels [14].

As in other viral infections, an increase in CRP is also possible in measles; it is especially noted that its value begins to rise in parallel with the appearance of the rash and that it increases significantly with developed complications such as pneumonia and encephalitis [5]. A study by Chilean researchers, which included 72 children, proved that the value of CRP can increase in the classic form of measles, but that it increases more significantly in complicated forms of measles and that its value is essential to determine in order to detect the onset of late complications in time [6]. A study conducted on 253 children with measles hospitalized in Daejeon St. Mary’s Hospital, Korea, determined that CRP values above 50 mg/L can indicate severe forms of the disease and complications [15]. In a study of 249 children, Italian researchers showed that the risk factors for severe outcomes in measles can be predicted by monitoring the CRP value without depending on the children’s age or premorbid condition [16].

It has been proven that there is severe immune dysfunction in the lungs during measles infection, with a loss of dendritic, CD4+, and NK+ cells and deficient cytokine production which is associated with viral infection [17]. This observation may be related to the fact that CRP regulates the generation and actions of dendritic cells in the periphery, which limits the activation of auto-reactive T cells and promotes the number and generation of myeloid-derived suppressor cells that are believed to suppress T cell proliferation [18]. An increase in serum CRP values might be, in some instances, a mechanism through which a body tries to decrease certain neutrophile functions, e.g., chemotaxis [19]. These effects of CRP could potentially be observed in our patients as well, in which the values of neutrophils were more or less within the range (Table 2); and even with pneumonia and measles, the patients had a similar extent of fever and decrease in blood O_2_ saturation (Table 3).

Recently, there has been a significant decrease in vaccination coverage, and there has been an increase in the number of patients with potential respiratory impairment during measles, such as various clinical–radiological findings ranging from interstitial pneumonia to bronchiolitis-like pictures; it is necessary to make an early respiratory assessment. The nature of the clinical presentation of measles and the possibility of rapid respiratory deterioration requires the determination of parameters that would timely indicate the development of respiratory complications in measles [20]. In the current outbreak, it was shown in our patients that the physical findings or subjective complaints of the patient at the first or follow-up examination do not necessarily indicate the development of complications, e.g., dyspneic complaints without the development of respiratory insufficiency or, conversely, the absence of dyspneic complaints and the occurrence of respiratory insufficiency; the development of viral pneumonia in patients without a cough. From that, it can be apprehended that it is helpful to monitor the patient more often (in our case, every three days) and monitor the extent of the CRP changes compared to the control value obtained during hospitalization. Our results showed that with an increase in the extent of pneumonia, a CRP increases severalfold as well (Table 4). This suggests that a severalfold increase in CRP, compared to the baseline, in patients with still clinically unmanifest complications can be indicative of a quick progression to a more severe life-threatening condition.

What is interesting to mention is that the chest X-ray changes in patients with measles do not correlate with the magnitude of CRP changes (Table 5). Interestingly, in patients with COVID-19, an increase in CRP above 29.8 mg/L might indicate the presence of lung infiltrates [21]. No correlation between CRP and X-ray changes has been analyzed up to now, and thus, this is the first study providing such findings where no correlation between an increase in CRP and radiological changes in the chest X-ray is found. Complicated measles is known to cause prolonged hospitalization, and among significant factors influencing the duration of hospitalization are the pulmonary condensations visible on X-ray [22].

## 5. Conclusions

Examining the changes in CRP serum values in patients with measles hospitalized at the Clinic for Infectious Diseases, University Clinical Center, Niš, we detected an association between the symptom severity (pneumonia category) and not with the changes detected on the chest X-ray. Thus, the obtained results point to a general predictive usefulness of serum CRP since its increase, compared to a baseline, might be indicative of the development of an underlying pulmonary complication, which is still clinically unmanifested.

## Figures and Tables

**Table 1:** Study age and disease duration-related parameters.

	Mean ± SD	Minimum	Maximum
*Admitted patients with measles (n = 207)*			
Age	36.3 ± 10.7	15	71
Total disease duration (days)	12.7 ± 4.7	1	33
Hospitalization time (days)	7.0 ± 4.3	5	24
Prehospital period with symptoms (days)	5.1 ± 2.0	1	14
*Patients with measles that did not develope pneumonia (n = 109)*			
Age	37 ± 10.6	16	76
Total disease duration (days)	12.5 ± 4.6	1	31
Hospitalization time (days)	6.3 ± 3.2	2	18
Prehospital period with symptoms (days)	4 ± 1.5	2	7
*Patients with measles who developed pneumonia (n = 98)*			
Age	37 ± 10.2	15	71
Total disease duration (days)	13.2 ± 4.9	2	33
Hospitalization time (days)	7.1 ± 4.2	7	24
Prehospital period with symptoms (days)	4.6 ± 1.6	3	8

**Table 2:** Blood and serum parameters in hospitalized patients with morbilli virus infection and those with pneumonia.

*Hospitalized patients with measles (n = 207)*	
*Blood cell analyses*	
Maximum WBC count (×10^5^)	6.1 ± 2.9
Neutrophile percent abundance	74.1 ± 10.2
*Serum biochemical analyses*	
LDH (U/L)	960 ± 157
CRP before hospitalization (mg/L)	32.4 ± 21.7
CRP during hospitalization (mg/L)	61.8 ± 27
*Patients with measles who did not develope pneumonia (n = 109)*	
*Blood cell analyses*	
Maximum WBC count (×10^5^)	6.8 ± 2.5
Neutrophile percent abundance	71.4 ± 17.1
*Serum biochemical analyses*	
LDH (U/L)	917 ± 394
CRP before hospitalization (mg/L)	27.5 ± 12.8
CRP during hospitalization (mg/L)	56.1 ± 10.4
*Patients with measles who developed pneumonia (n = 98)*	
*Blood cell analyses*	
Maximum WBC count (×10^5^)	6.0 ± 2.1
Neutrophile percent abundance	79.6 ± 12.4
*Serum biochemical analyses*	
LDH (U/L)	1046 ± 345
CRP before hospitalization (mg/L)	31.3 ± 8.5
CRP during hospitalization (mg/L)	72.2 ± 12.6

**Table 3:** Clinical parameters during hospitalization.

	*Total Sample*			*Patients with Pneumonia*			*Patients without Pneumonia*		
*Measured Parameter*	Mean ± SD	Min	Max	Mean ± SD	Min	Max	Mean ± SD	Min	Max
Maximal fever (°C)	39.2 ± 0.7	37.2	41	39.1 ± 0.1	37.4	41	39.2 ± 0.3	37.2	40.5
Fever duration (days)	7.5 ± 2.8	2	17	7.6 ± 2.2	4	15	7.8 ± 5.4	2	17
Blood O_2_ saturation (%)	94.1 ± 5.2	41	100	92.1 ± 7.2	41	100	95.2 ± 3.1	84	100
*Observed parameter*	Number (*n*)	Percent (%)		Number (*n*)	Percent (%)		Number (*n*)	Percent (%)	
Positive chest X-ray findings	98	47.3		98	100		109	0	
Dry cough	171	82.6		88	89.7		83	66.9	
Asphyxiation	95	45.9		87	88.8		8	7.3	
Sore throat	87	42		36	36.7		51	46.8	
Koplik spots	21	10.1		8	8.1		13	1.2	
Enanthem	15	7.2		8	8.1		7	6.4	
*Comorbidity*	Number (*n*)	Percent (%)		Number (*n*)	Percent (%)		Number (*n*)	Percent (%)	
Total number of comorbidity *	34	16.5		17	17.3		17	15.6	
Hypertension	6	2.9		3	3.1		3	2.7	
Chronic obstructive pulmonary disorder	4	1.9		2	2.0		2	1.8	
Myocardial infarct	4	1.9		-	-		4	3.7	
Pregnancy	4	1.9		2	2.0		2	1.8	
Congenital mental disorder	3	1.4		-	-		3	2.7	
Asthma	2	0.9		2	2.0		-	-	
Other **	10	4.8		10	10.2		-	-	

* Some patients had more than one comorbidity: ** single comorbidity such as hypothyroidism, psoriasis, rheumatoid arthritis, etc.

**Table 4:** Association between changes in CRP levels and the severity of pneumonia.

		Category of Pneumonia				χ^2^	*p*
		1	2	3	4		
Changes in CRP	1	0 (0%)	1 (3.6%)	1 (10%)	0 (0%)	45.083	0.0001
	2	33 (97.1%)	9 (32.1%)	2 (20%)	0 (0%)		
	3	0 (0%)	10 (35.7%)	3 (30%)	1 (25%)		
	4	1 (2.9%)	8 (28.6%)	4 (40%)	3 (75%)		

**Table 5:** Association between changes in CRP and chest X-ray radiographic changes.

		X-ray Changes		Total	χ^2^	*p*
		No	Yes			
Changes in CRP	1	0 (0%)	2 (2.7%)	2 (2.63%)	2.271	0.518
	2	3 (100%)	41 (56.2%)	44 (57.89%)		
	3	0 (0%)	14 (19.2%)	14 (18.42%)		
	4	0 (0%)	16 (21.9%)	16 (21.05%)		

## Data Availability

Data will be available from the corresponding author upon reasonable request.
